# Endoplasmic Reticulum Stress and Pathogenesis of Vascular Calcification

**DOI:** 10.3389/fcvm.2022.918056

**Published:** 2022-06-16

**Authors:** Zhenqi Rao, Yidan Zheng, Li Xu, Zihao Wang, Ying Zhou, Ming Chen, Nianguo Dong, Zhejun Cai, Fei Li

**Affiliations:** ^1^Department of Cardiovascular Surgery, Union Hospital, Tongji Medical College, Huazhong University of Science and Technology, Wuhan, China; ^2^Basic Medical School, Tongji Medical College, Huazhong University of Science and Technology, Wuhan, China; ^3^Department of Cardiology, The Second Affiliated Hospital, Zhejiang University School of Medicine, Hangzhou, China

**Keywords:** vascular calcification, endoplasmic reticulum stress, unfolded protein response, chronic kidney disease, atherosclerosis, diabetes

## Abstract

Vascular calcification (VC) is characterized by calcium phosphate deposition in blood vessel walls and is associated with many diseases, as well as increased cardiovascular morbidity and mortality. However, the molecular mechanisms underlying of VC development and pathogenesis are not fully understood, thus impeding the design of molecular-targeted therapy for VC. Recently, several studies have shown that endoplasmic reticulum (ER) stress can exacerbate VC. The ER is an intracellular membranous organelle involved in the synthesis, folding, maturation, and post-translational modification of secretory and transmembrane proteins. ER stress (ERS) occurs when unfolded/misfolded proteins accumulate after a disturbance in the ER environment. Therefore, downregulation of pathological ERS may attenuate VC. This review summarizes the relationship between ERS and VC, focusing on how ERS regulates the development of VC by promoting osteogenic transformation, inflammation, autophagy, and apoptosis, with particular interest in the molecular mechanisms occurring in various vascular cells. We also discuss, the therapeutic effects of ERS inhibition on the progress of diseases associated with VC are detailed.

## Introduction

Vascular calcification (VC) commonly occurs with aging, atherosclerosis, diabetes, and chronic kidney disease (CKD) owing to accumulation of apatite calcium salts in the media and/or intima of arteries (1). Based on the location of hydroxyapatite, three main types of VC have been reported, namely, intimal calcification, Mönckeberg medial arterial calcification, and valvular calcification (2). Intimal calcification is closely associated with lipid deposits, inflammatory cell infiltration, atherosclerosis, and atherosclerotic plaque rupture (3). Medial calcification occurs when smooth muscle cells transform into osteoblast-like cells. This transformation is associated with various genes, including bone morphogenetic protein-2 (*BMP2*), MSH homeobox 2 (*MSX2*), and alkaline phosphatase (*ALP*; 4). Valvular calcification occurs in aortic valves as a result of long-term mechanical stress and the effects of proinflammatory cytokines, which can cause aortic stenosis.

The endoplasmic reticulum (ER) is an intracellular organelle with important roles in protein folding and maturation, lipid biosynthesis, and intracellular calcium homeostasis. However, the capacity of the ER protein maturation machinery can become overwhelmed in certain physiological or pathological conditions, leading to the accumulation of defective or superfluous proteins and ER stress (ERS; 5). Over the past two decades, ERS has been widely recognized as an important mechanism implicated in the development of several human diseases. Moreover, numerous studies have shown that ERS contributes to VC through various mechanisms in vascular cells. VC research is currently focused on the intima and tunica media; involvement of the former is observed in atherosclerosis and that of the latter in degenerative vascular diseases, such as CKD and diabetes; however, no effective clinical therapy is available for VC.

In this review, we summarize the roles of ERS in the initiation and progression of VC. Furthermore, we discuss the current understanding of how ERS can promote VC through various mechanisms involving vascular smooth muscle cells (VSMCs), vascular endothelial cells (VECs), and immune cells, leading to an increase in the unfolded protein response (UPR). Finally, we evaluate emerging therapeutic strategies to target VC-associated ERS.

## Key Players in the Unfolded Protein Response

**FIGURE 1:**
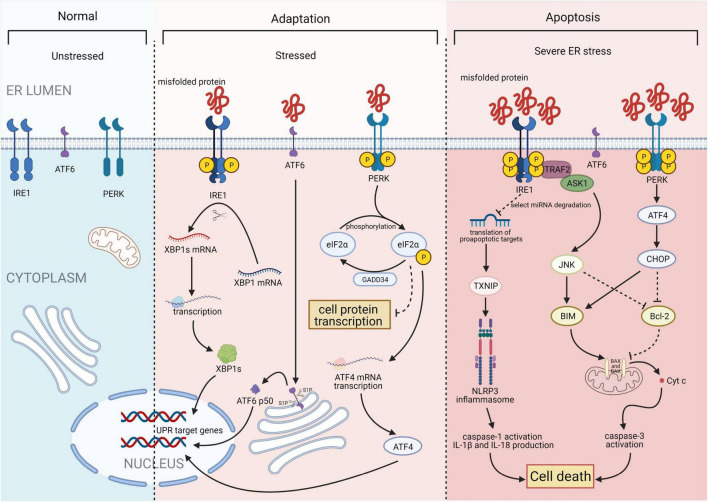
Three major states of endoplasmic reticulum stress (ERS): unstressed, stressed, and severe ERS. In the absence of stress, PKR-like ER kinase (PERK), activating transcription factor 6 (ATF6), and inositol-requiring enzyme 1 (IRE1) are maintained in an inactive state. Upon activation, the IRE1 endoribonuclease domain specifically recognizes and cleaves a 26-base fragment from the X-box binding protein 1 (XBP1) mRNA transcript, resulting in XBP1s. PERK phosphorylates the alpha subunit of eukaryotic translation initiation factor 2 (eIF2α), that leads to a downregulation of overall mRNA translation, which reduces the accumulation of unfolded proteins. Phosphorylation of eIF2α also induces the translation of ATF4. ATF6 will be cleaved by two Golgi resident proteases to release its N-terminal transcriptionally active 50-kDa fragment. Thereafter, the 50-kDa N-terminus protein will translocate into the nucleus to activate transcription. Finally, after activation of ATF6, IRE1, and PERK, the unfolded protein response (UPR) is constitutively activated. Under severe ERS, IRE1 forms a complex with tumor necrosis factor receptor-associated factor 2 (TRAF2) and apoptosis signal-regulating kinase 1 (ASK1) to activate c-Jun N-terminal kinase (JNK) and the caspase family. This process activates the NACHT, LRR, and PYD domain-containing protein 3 (NLRP3) inflammasome. ATF4 and C/EBP homologous protein (CHOP) synergistically induce the expression of multiple genes involved in apoptosis, autophagy, and antioxidant responses, which all lead to cell death. Figures were created using BioRender.com.

The UPR is a highly conserved mechanism mediated by three ER transmembrane sensor proteins: PKR-like ER kinase (PERK), activating transcription factor 6 (ATF6), and inositol-requiring enzyme 1 (IRE1; Figure 1). In the absence of stress, these proteins are maintained in an inactive state by the binding of their luminal domains with the ER intraluminal glucose-regulated protein 78 (GRP78; 6). ERS conditions activate these proteins and increase the binding of GRP78 to misfolded or unfolded proteins. This results in the dissociation of GRP78 from ATF6, IRE1, and PERK and the subsequent activation of UPR signaling (7).

### Inositol-Requiring Enzyme 1

Inositol-requiring enzyme 1, the most conserved UPR transducer, is a transmembrane protein responsible for protein kinase and endoribonuclease activity (8, 9). IRE1 homodimerizes and is transphosphorylated upon dissociation from GRP78, facilitating subsequent allosteric activation of its C-terminal endoribonuclease domain (10, 11). This domain specifically recognizes and cleaves a 26-base fragment from the mRNA transcript of the X-box binding protein 1 (XBP1). The 26-base transcript fragment is subsequently translated into spliced XBP1s (XBP1s), which act as a transcription factors by inducing the expression of genes involved in protein folding, autophagy, and ER-associated degradation to help maintain ER homeostasis (12). In addition, IRE1 forms a complex with tumor necrosis factor receptor-associated factor 2 and apoptosis signal-regulating kinase 1, leading to the activation of c-Jun N-terminal kinases and caspase proteases, which promote apoptosis (13).

### PKR-Like Endoplasmic Reticulum Kinase

PKR-like ER kinase, a type I ER-resident protein kinase, is activated through autophosphorylation and homodimerization after dissociating from GRP78 (14). Once activated, PERK phosphorylates the alpha subunit of eukaryotic translation initiation factor (eIF) 2α, leading to downregulation of overall protein synthesis. This reduces the accumulation of unfolded proteins. However, phosphorylation of eIF2α also induces the translation of ATF4 (15), which stimulates the expression of C/EBP homologous protein (CHOP). Subsequently, ATF4 and CHOP synergistically induce the expression of genes involved in apoptosis, autophagy, and antioxidant response (16).

### Activating Transcription Factor 6

Activating transcription factor 6 is a type II ER transmembrane protein with a basic leucine zipper transcription activation domain at the N-terminus. The ATF6 C-terminus is localized in the ER cavity. ATF6 has multiple GRP78 binding sites and two Golgi positioning signals. GRP78 dissociates from the luminal domain of ATF6 in response to ERS, exposing two Golgi localization signals and causing ATF6 translocation to the Golgi (17). Following translocation, ATF6 is cleaved by site-1 protease and site-2 protease to release its active N-terminal fragment (18). The N-terminus protein then binds to the ATF/cyclic adenosine monophosphate response element and ERS response element and subsequently migrates into the nucleus to activate transcription. Furthermore, to reduce unfolded protein accumulation, ATF6 regulates the transcription of various genes, such as those encoding ER chaperones and protein folding enzymes. This process is mediated by activating specific UPR-related genes (including *XBP1*) and three main branches of the UPR (including *ATF4*, *XBP1*, and *ATF6*; 12).

## Vascular Calcification

Vascular calcification occurs when calcium phosphate crystals (hydroxyapatite) accumulate in the media and/or intima of vessel walls and is strongly correlated with cardiovascular mortality in patients with CKD, atherosclerosis, and diabetes (19, 20). Hence, delaying and reversing VC can theoretically reduce the case fatality rate of these high-risk groups. However, the specific mechanisms underlying VC development and pathogenesis are not fully understood.

### Predisposition of Vascular Calcification

Multiple factors contribute to the occurrence of VC. Physiological calcification is a normal process occurring in bones and teeth; however, its occurrence is closely associated with aging, advanced atherosclerosis, diabetes mellitus, and CKD (21). Moreover, numerous risk factors, including calcium and phosphorus metabolism disorder, inflammation, oxidative stress, apoptosis, autophagy, and aging, can also contribute to VC (22).

### Mechanisms of Vascular Calcification

In the past decade, VC was defined as a passive, degenerative condition. However, more recent studies have suggested that VC is a highly regulated process resulting from multiple factors acting together over a certain period of time. For example, the transformation of smooth muscle cells from a contractile phenotype to an osteoblast-like phenotype, together with extracellular matrix remodeling, apoptosis, and elevated calcium and phosphorus levels, is involved in the development of VC. Under the action of calcification-stimulating factors, the expression levels of osteoblast cell markers, including Runt-related transcription factor 2 (Runx2), become increased, as does the activity of the transcription factor core binding factor alpha1 and the expression of genes containing the core binding factor alpha1 binding site (i.e., osteopontin, osteocalcin, and ALP; 23). By contrast, the expression levels of smooth muscle cell markers, such as SM22-α and smooth muscle α-actin, are decreased, thereby promoting the transformation of smooth muscle cells from a contractile phenotype to an osteoblast cell phenotype (23). Moreover, disorder of calcium (Ca^2+^) and phosphorus (Pi) metabolism promotes VC. That is, high Ca^2+^ and Pi concentrations can promote the expression of BMP2, Runx2, MSX2, and osteocalcin in VSMCs, thus promoting osteogenic-like differentiation of VSMCs. In addition, imbalanced Ca^2+^ and Pi concentrations can cause accumulation of Ca^2+^ in VSMCs, thereby promoting the release and mineralization of matrix vesicles as well as the apoptosis of VSMCs (24, 25). However, despite the abundance of research on the various risk factors for VC, including hyperphosphatemia, hypercalcemia, oxidative stress, inflammation, and apoptosis, there is a dearth of information regarding the associated regulatory pathways and molecules. Therefore, as a calcium reservoir, the ER has high value in the field of VC research.

## Endoplasmic Reticulum Stress and Vascular Calcification

Numerous recent studies have shown that ERS can regulate VC through various mechanisms in vascular cells (Table 1; 26).

**TABLE 1:** Reference table to the specific cell type, signaling pathway leading to ERS, and the mechanism of VCd LDL.

Specific cell-type	Pathway	Biological effect	References
Vascular smooth muscle cell	IRE1	Osteogenic differentiation	(36)
	ATF4	Osteogenic differentiation	(37, 38)
		VSMC apoptosis	(40)
		VSMC autophagy	(47)
	ATF6	Osteogenic differentiation	(35)
	Caspase-12	VSMC apoptosis	(41)
	Matrix vesicles (MVs)	VSMC autophagy	(48, 49)
VEC	IRE1-EndMT	Osteogenic differentiation	(58)
	IRE1	VEC apoptosis	(65)
	ATF6	VEC apoptosis	(66)
Macrophages	ATF4	Macrophage-derived foam cell formation and apoptosis	(80)
	ATF6, p-IRE1α	Alleviated inflammation of macrophages	(94)
Inflammasomes	ATF4	Subsequent inflammation triggered by NLRP3	(87)

### Endoplasmic Reticulum Stress in Vascular Smooth Muscle Cells

**FIGURE 2:**
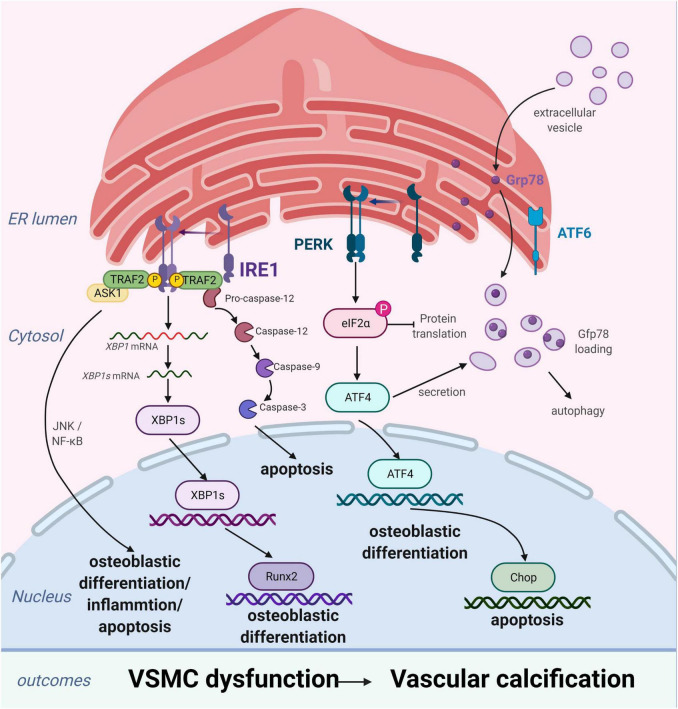
Endoplasmic reticulum stress (ERS) promotes vascular calcification (VC) by inducing the osteogenic differentiation, autophagy, and apoptosis of vascular smooth muscle cells (VSMCs). In response to ERS, VSMCs differentiate into calcifying VSMCs *via* multiple mechanisms, including osteogenic differentiation, apoptosis, and autophagy. Inositol-requiring enzyme 1 (IRE1) can promote osteoblastic differentiation *via* nuclear factor kappa B (NF-κB), the IRE1α-XBP1 axis, and RUNX2 signaling pathways and aggravate apoptosis by the IRE1α-ASK-JNK and caspase-12 pathways. PKR-like ER kinase (PERK) induces apoptosis *via* the PERK-eIF2α-CHOP signaling pathway and osteogenic differentiation *via* ATF4 activation. Extracellular vesicles, induced by increased expression of Grp78 and ATF4, attract inflammatory cells and induce VC. Figures were created using BioRender.com.

Vascular smooth muscle cells are the most abundant cell type in the arterial vessel wall, and play a key role in regulating atherosclerotic plaque formation and VC (27). In response to ERS, VSMCs differentiate into calcified vascular cells through multiple mechanisms, including osteogenic differentiation (28), apoptosis (29), autophagy induction (30), cellular senescence (31), and oxidative stress (32). Many of these processes also occur with bone formation. Importantly, ERS and the UPR are crucial for bone development. All three branches of the UPR are activated during bone formation to regulate the expression of osteogenic genes. ERS is strongly associated with VC, particularly in VSMCs (Figure 2; 33, 34).

#### Endoplasmic Reticulum Stress Promotes Osteogenic Differentiation of Vascular Smooth Muscle Cells

The process of VC involves vascular cells, mainly VSMCs, undergoing osteogenic processes that resemble osteoblast formation, such as osteogenic differentiation, matrix maturation, and matrix mineralization stages. The osteogenic differentiation of VSMCs induced by ERS plays an important role in VC development. This is supported by studies demonstrating that ERS can promote osteogenic differentiation of VSMCs through three pathways, namely IRE1-XBP1, PERK-eIF2α-ATF4, and ATF6. Downregulation of the expression of genes involved in the UPR, including *HSPA5*, *XBP1*, *ATF4*, *DDIT3*, and *ATF6*, has been shown to suppress osteogenic gene expression and mineralization of VSMCs (35). Runx2 is a key transcription factor involved in osteoblast differentiation and its expression can be directly regulated by XBP1 (36). ATF4 is a key transcription factor involved in osteoblastogenesis and ERS-induced apoptosis. Notably, ATF4 deficiency has been reported to inhibits osteogenic differentiation and calcification of VSMCs, both *in vitro* and *in vivo* (37, 38).

#### Endoplasmic Reticulum Stress Promotes Vascular Smooth Muscle Cell Apoptosis

Apoptosis is closely related to calcification. Calcified VSMCs are prone to apoptosis, and apoptosis can in turn promote calcification. VSMC apoptosis provides a suitable microenvironment for the nucleation of hydroxyapatite crystals, which play a key role in the initiation of VC. Moreover, VSMC apoptosis directly affects the morphology and structure of advanced atherosclerosis and plaque stability (39). VSMC apoptosis process can be activated by ERS through three apoptotic pathways: the IRE1α-ASK-JNK, PERK-eIF2α-CHOP signaling, and caspase-12 pathways. CHOP, an ERS-specific transcription factor, is activated by the PERK-eIF2α-ATF4 pathway and induces apoptosis by decreasing the expression of the anti-apoptotic protein, B-cell lymphoma 2. Shiozaki et al. found that transgenic mice with SMC-specific CHOP expression develop severe vascular apoptosis and medial calcification with CKD (40). They further demonstrated that the cyclin-dependent kinase 9 (CDK9)-cyclin T1 complex mediates VC through CHOP induction and phosphorylation-mediated ATF4 activation. Caspase-12, activated exclusively by ERS, activates caspase-9 directly, which subsequently activates caspase-3, resulting in apoptosis (41). Shi et al. found that the metabolic hormone fibroblast growth factor 21 (FGF21) inhibits VC progression by alleviating ERS-mediated apoptosis in rats. The caspase-12 pathway, but not the phospho-JNK-JNK pathway, is involved in FGF21 expression (34). Moreover, expression levels of JNK, which plays a key role in cell differentiation, inflammation, and apoptosis, did not change with ERS-induced apoptosis in a rat model of periodontitis and VC (42). Therefore, the role of the IRE1α-ASK-JNK pathway in VC development needs further investigation.

#### Crosstalk Between Endoplasmic Reticulum Stress and Autophagy in Vascular Smooth Muscle Cells

Autophagy is a catabolic and tightly regulated subcellular process in which long-lived proteins and damaged organelles are degraded by lysosomes. Autophagy can regulate endothelial cell homeostasis, VSMC phenotype transition, and Ca^2+^ homeostasis in VSMCs (2). Emerging evidence has demonstrated that autophagy directly protects against VC. Morciano et al. found that the autophagy and mitochondrial phagocytosis levels of caveolins increased and that rapamycin can enhance the calcification phenotype by promoting autophagy *in vitro* (43). In addition, crosstalk among endosomes, dysfunctional mitochondria, autophagic vesicles, and Ca^2+^- and Pi-enriched matrix vesicles (MVs) may underlie the pathogenesis of VC. Autophagy is an adaptive response that protects against phosphate-induced VSMC calcification by regulating apoptosis and releasing mineralizing MVs from VSMCs (44–46). Moreover, autophagosomes are formed by shedding the double-layer membrane of the ribosome free attachment area of the rough ER and wrapping some cytoplasmic and intracellular organelles, proteins, and other components that need to be degraded. Therefore, the ER plays an indispensable role in autophagy regulation. ERS activates autophagy through the UPR and calcium-mediated signaling cascade pathway. However, the associations of ERS, autophagy, and VC remain poorly understood. Furmanik et al. reported that ERS mediates VSMC calcification *via* increased release of extracellular vesicles, induced by increased expression of GRP78 and ATF4 (47). However, another study on VC revealed that autophagy results in the release of MVs, which attract inflammatory cells and induce VC (48, 49). Therefore, further studies of the mechanisms linking ERS and autophagy in VSMCs are warranted.

### Endoplasmic Reticulum Stress in Vascular Endothelial Cells

**FIGURE 3:**
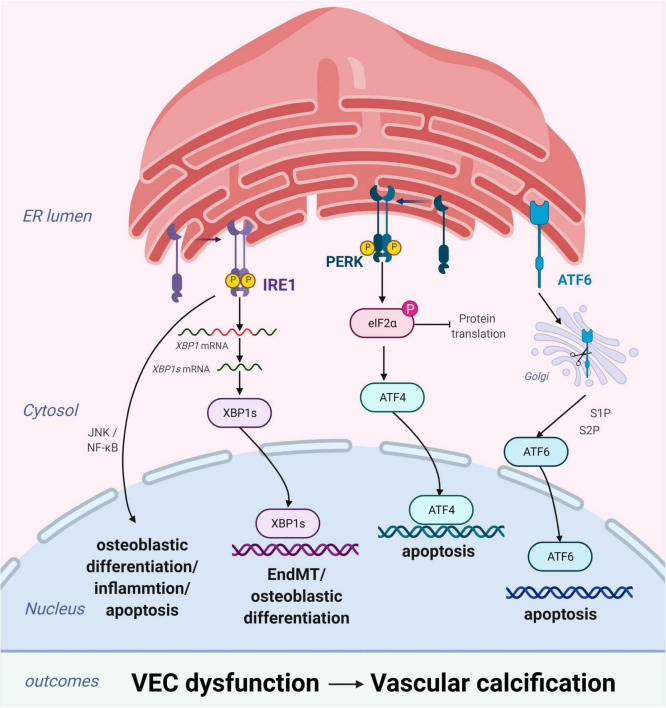
Endoplasmic reticulum stress (ERS) promotes vascular calcification (VC) by inducing the osteogenic differentiation and apoptosis of vascular endothelial cells (VECs). In response to ERS, inositol-requiring enzyme 1 (IRE1) can aggravate EndMT through the IRE1α-XBP1 axis, promote osteoblastic differentiation *via* the nuclear factor kappa B (NF-κB) signaling pathway and the IRE1α-XBP1 axis, and induce apoptosis *via* the IRE1α-ASK-JNK pathway. PKR-like ER kinase (PERK) promotes apoptosis *via* the PERK-eIF2α-ATF4 signaling pathway. High expression of active ATF6 may exacerbate VEC apoptosis through the mitochondrial apoptotic pathway. Figures were created using BioRender.com.

Vascular endothelial cells, which form a biological barrier that controls the passage of immune cells and biomolecules between the vascular walls and mediates physiological functions, are the main components of the vascular intima. During homeostatic conditions, endothelial cells maintain microvascular integrity and exert vasodilatory, anti-inflammatory, and antithrombotic activities (50). However, under various pathological conditions, these biological functions become compromised (51) and may lead to VC through osteogenic differentiation, endothelial microparticles, or cytokines. ERS plays an important role in these changes primarily by inducing osteogenic differentiation and apoptosis of VECs (Figure 3).

#### Endoplasmic Reticulum Stress Promotes Osteogenic Differentiation of Vascular Endothelial Cells

Similar to VSMCs, VECs promote VC by inducing osteogenic differentiation (52). Changes in VEC physiological homeostasis promotes osteoblastic differentiation *via* the extracellular signal-regulated protein kinase 1/2 and nuclear factor kappa B (NF-κB) signaling pathways, leading to VC (53). Moreover, studies have substantiated that endothelial-to-mesenchymal transition (EndMT) plays a critical role in the osteogenic differentiation of VECs (54–56). EndMT is a process through which endothelial lineage cells lose cell polarity, acquire migratory and aggressive characteristics, and differentiate into mesenchymal stem cells. Moreover, the EndMT gives VECs the potential for osteogenesis differentiation in different physiological states. Yao et al. found that reducing the EndMT by knocking out serine protease or Sox2 in *in vitro* experiments improves VC (57). EndMT occurs when endothelial cells acquire mesenchymal and stem-cell-like characteristics and is closely related to ERS (58, 59). ERS can aggravate EndMT *via* the IRE1α-XBP1 axis and thereby contribute to VC (58).

#### Endoplasmic Reticulum Stress Promotes Vascular Endothelial Cell Apoptosis

Apoptosis of endothelial cells plays a pivotal role in the development of VC. Increased endothelial cell apoptosis has been observed in the atherosclerosis-prone regions of the vasculature (60) and in the endothelium of human atherosclerotic plaques (61). Under homeostatic conditions, clearance of apoptotic cells occurs in the absence of immune activation and is mediated by various phagocytic cells (7, 62). However, dysregulation of apoptotic cell clearance can lead to secondary necrosis and release of proinflammatory intracellular contents (62). Moreover, this can promote the development of chronic inflammatory diseases, such as atherosclerosis (63). ERS-induced VEC apoptosis plays an important role in the pathogenesis and development of several vascular diseases (64, 65). Recent studies have demonstrated that high expression of active ATF6 may exacerbate VEC apoptosis through the mitochondrial apoptotic pathway (66). Similarly, the activation of IRE1α may enhance VEC apoptosis *via* the pro-apoptotic molecule JNK and the p38-mitogen-activated protein kinase pathway (65). Furthermore, VEC apoptosis changes the balance between pro-apoptotic and anti-apoptotic proteins of the Bcl-2 family, leading to VC (67).

### Endoplasmic Reticulum Stress and Inflammation

Vascular calcification is associated with the development of chronic inflammation, as inflammatory cell infiltration has been detected in all stages of VC (68–71). Therefore, in addition to VSMCs and VECs, VC therapeutic strategies targeting inflammation warrant further investigation. ERS is associated with various pathological conditions linked to chronic inflammation (72, 73). Studies indicate that ERS can trigger inflammatory pathways and proinflammatory stimuli, such as Toll-like receptor ligands, reactive oxygen species, and cytokines. These proinflammatory signals can then initiate ERS and result in UPR activation, which further amplifies inflammatory responses (74). Collectively, these processes increase the chances of developing VC (75).

#### Endoplasmic Reticulum Stress in Macrophages

Macrophages are central effectors of innate immunity and play a crucial role in VC development. Studies have shown that activated macrophages contribute to VC by differentiating into osteoclasts (76) and secreting inflammatory factors (77). ERS induces an inflammatory response in macrophages *via* IRE1α-XBP1 (78) and PERK-ATF4 (79). Yao et al. found that D4F, an apoA-I mimetic peptide, can alleviate macrophage-derived foam cell formation and apoptosis by inhibiting CD36-mediated ox-LDL uptake and subsequent activation of the ERS-CHOP pathway (80). Ren et al. found that intermedin1-53, a cardiovascular protective peptide, protects against homocysteine-promoted atherosclerotic calcification in ApoE^–/–^ mice by inhibiting ERS markers in rat VSMCs and mouse peritoneal macrophages (81). Further investigation is needed to better understand the role of ERS in macrophage-induced VC.

#### Endoplasmic Reticulum Stress and Inflammasomes

The NACHT, LRR, and PYD domains-containing protein 3 (NLRP3) inflammasome is an essential component of the innate immune system and can induce the secretion of the proinflammatory cytokine interleukin 1 beta (IL-1β) in a caspase-1-dependent manner (82, 83). Moreover, IL-1β can activate the secretion of the receptor activator of NF-κB ligand, which promotes the formation of osteoclasts, leading to VC (84, 85). ERS stimulates the NLRP3 inflammasome activation through oxidative stress, NF-κB activation, and calcium homeostasis (86). Ren et al. found that intermedin may attenuate the progression of atherosclerotic lesions and plaque susceptibility by inhibiting ERS-CHOP-mediated macrophage apoptosis and subsequent inflammation triggered by NLRP3 both *in vivo* and *in vitro* (87). Therefore, further studies are needed to elucidate the mechanisms of ERS-mediated and NLRP3 inflammasome-mediated VC.

## Endoplasmic Reticulum Stress-Mediated Regulation of Vascular Calcification in Diseases

It is important to identify effective treatments for the globally prevalent metabolic disorders, such as aging, CKD, diabetes, and atherosclerosis-related VC (88). However, no effective clinical therapy is currently available. ERS is a key feature of metabolic disorders. Hence, herein we aim to identify new therapeutic targets for the treatment of VC by discussing the effects of ERS in different diseases.

### Chronic Kidney Disease

Although there have been recent improvements in the systemic management of CKD, cardiovascular disease remains a leading cause of death in patients with CKD (89, 90). VC is a common complication in patients with CKD and is associated with increased cardiovascular disease-related mortality. Recent evidence suggests that ERS is the primary cause of VC in CKD *via* several pathways. Tumor necrosis factor α induces the PERK-eIF2α-ATF4-CHOP axis of the ERS response, leading to CKD-associated VC (91). Other positive regulators of the PERK-eIF2α-ATF4-CHOP axis of the ERS response in VSMCs include high phosphate levels, oxidized lipids, *BMP2*, and basic fibroblast growth factor (92, 93). Furthermore, oxysterol accumulation in the ER induces ERS and activates CKD-dependent VC *via* the PERK-eIF2α-ATF4-CHOP pathway. Oxysterol-mediated ERS can be reduced by ezetimibe-simvastatin combination therapy, thereby attenuating CKD-dependent vascular diseases (94). In addition, ATF4 activity, which is activated by the CDK9-cyclin T1 complex during ERS, can lead to VC in CKD. Moreover, inhibition of the cyclin T1-CDK9-CHOP pathway may decrease ERS-induced CHOP expression and CKD-dependent VC (40).

### Atherosclerosis

Atherosclerosis is a chronic inflammatory disease characterized by the progressive accumulation of lipids and plaques in arteries (95). Pathological conditions, such as inflammation, oxidized lipids, and metabolic stress, can activate ERS (96, 97). The UPR is chronically activated in atherosclerotic lesion cells, particularly advanced lesional macrophages, and endothelial cells. Tabas found that ERS is a significant cause of apoptosis of endothelial cells and macrophages in advanced lesions (60). In addition, Oh et al. reported that suppression of macrophage ERS can lead to polarization of differentiated M2 macrophages toward an M1 phenotype and can subsequently suppressed foam cell formation (98). Furthermore, intermedin1-53 protects against homocysteine-related atherosclerotic calcification in Apoe^–/–^ mice by inhibiting ERS (81). These studies indicate that ERS plays a significant role in atherosclerosis and that inhibiting ERS can alleviate the pathological damage associated with atherosclerotic calcification.

### Diabetes

Despite improvements in CVD treatment over the past few decades, diabetes remains a significantly independent cardiovascular risk factor (61, 99). Therefore, reducing adverse events caused by diabetes-induced CVD is a clinical challenge (100). Diabetes-related VC presents with disturbed vessel wall homeostasis, endothelial dysfunction, and phenotypic switching of VSMCs (101–103). High glucose levels trigger apoptosis and phenotypic transformation of VSMCs (in the presence of ERS). In addition, compared with continued high glucose conditions, increased glycemic variability is more strongly associated with VC (104). Chronic exposure of VSMCs to high glucose conditions can exacerbate inflammation and calcification through the induction of CD36 scavenger receptors (71). Moreover, CD36 signaling may contribute to diabetic atherosclerosis *via* ERS induction. Collectively, these findings suggest that ERS crucially contributes to diabetes; however, further investigation is warranted.

## Current Clinical Trials Assessing Endoplasmic Reticulum Stress-Related Vascular Calcification Treatment

Endoplasmic reticulum stress plays a key pathological role in promoting the occurrence and development of CVDs. Over the past few decades, research has focused on the signaling proteins involved in ERS, resulting in the development of an increasing array of drugs (Table 2). This section aims to explain the fundamentals, value, and limitations of existing drugs targeting ERS.

**TABLE 2:** Table summarizing ERS blockade attempt in diseases and their main outcome on VC.

Blockade	Main outcome on VC	References
IMD1-53	Attenuates VSMC calcification in rats by inhibiting ERS through cAMP/PKA signaling	(105)
Sodium selenite	Suppresses apoptosis of calcifying VSMCs by inhibiting oxidative-stress-activated ERS	(106)
Cyclin T2 and cyclin K	Decreases ERS-induced CHOP expression and CKD-dependent VC	(40)
Spermidine	Ameliorates VSMC calcification through sirtuin 1-mediated inhibition of ERS	(109)
Stellate ganglion block (SGB)	Prevents the activation of ERS by inhibiting the sympathetic nervous system to regulate vascular dilation	(110)
Fibroblast growth factor 21 (FGF21)	Inhibits the progress of VC by alleviating ERS mediated apoptosis in rats	(34)
Death-associated protein kinase 3 (DAPK3)	Regulates VSMC calcification *via* AMPK-mediated ERS signaling	(111)
Ezetimibe-simvastatin	Attenuates CKD-dependent vascular diseases	(99)

Intermedin1-53, a paracrine/autocrine peptide in the vasculature, attenuates VSMC calcification in rats by inhibiting ERS through cyclic adenosine monophosphate-protein kinase A signaling (105). Sodium selenite may suppress apoptosis of calcifying VSMCs, in part, by inhibiting oxidative-stress-activated ERS (106). The CDK9-cyclin T1 complex, an essential component in ERS, mediates pro-apoptotic CHOP expression and VC by activating ATF4. Cyclin T2 and cyclin K inhibit CHOP induction by competitively binding CDK9. Hence, inhibition of the cyclin T1/CDK9-CHOP pathway may be a potential therapeutic strategy for VC treatment (107). Spermidine, an endogenously synthesized polyamine, has been shown to protect against CVD and extend lifespan (108). VSMC calcification has recently been shown to be ameliorated through sirtuin 1-mediated inhibition of ERS (109). Stellate ganglion block, which regulates vascular dilation through sympathetic blockade, is used to treat several CVDs. Recently, stellate ganglion block has been shown to prevent the activation of ERS by inhibiting sympathetic nervous activity (110). For the first time, FGF21 has been shown to reduce ERS-mediated VC progression in rats. Some studies suggest that FGF21 has the potential to regulate many metabolic diseases and CVDs because of its pleiotropic biological effects. Thus, FGF21 is a promising new therapeutic target for preventing and treating VC (34). Death-associated protein kinase 3 is involved in hypertension-related vascular remodeling and has been shown to regulate VSMC calcification *via* AMPK-mediated ERS signaling (111). Although these studies provide important translational insights into ERS-targeted prevention of VC, they present only basic experiments and therefore, clinical studies are needed. Notably, inappropriate alteration of ERS may also cause harm, as it is a key mechanism in body maintenance. Hence, the safety of ERS inhibitors requires further evaluation prior to their clinical application.

## Conclusion and Perspective

Despite various clinical prevention strategies, CVD remains a common complication of aging, atherosclerosis, hypertension, diabetes, and CKD. Accumulating evidence indicates that ERS can regulate the development of VC by promoting osteogenic transformation, inflammation, autophagy, and apoptosis, and by increasing the UPR. Although these studies comprehensively demonstrate that ERS inhibitors can ameliorate VC, they all have certain limitations. That is, most of these studies conducted basic experiments that did not fully nor accurately reflect the pathological changes in human diseases. Additionally, considering that the ERS-UPR pathway is ubiquitous in humans and sensitive to external stimuli, the repression of excessive ERS can promote cellular damage and lead to increased disease progression. Therefore, clinical trials are needed to validate the results of these studies; this may aid in the development of new therapies for VC.

## Author Contributions

ZR, LX, YDZ, ZW, YZ, MC, ND, ZC, and FL: conceptualization. All authors writing-original draft preparation, editing, and revising. FL and ZC: supervision.

## Conflict of Interest

The authors declare that the research was conducted in the absence of any commercial or financial relationships that could be construed as a potential conflict of interest.

## Publisher’s Note

All claims expressed in this article are solely those of the authors and do not necessarily represent those of their affiliated organizations, or those of the publisher, the editors and the reviewers. Any product that may be evaluated in this article, or claim that may be made by its manufacturer, is not guaranteed or endorsed by the publisher.
